# The Role of Teacher Autonomy Support on Students’ Academic Engagement and Resilience

**DOI:** 10.3389/fpsyg.2021.778581

**Published:** 2021-11-05

**Authors:** Qiangqiang Ma

**Affiliations:** School of Marxism, Jilin University of Finance and Economics, Changchun, China

**Keywords:** academic engagement, students’ resilience, teacher autonomy support, enthusiasm, educational achievement

## Abstract

Learners have internal motivational resources that, when maintained, can enhance engagement, enthusiasm, resilience, and success. Learner engagement in educational tasks is a remarkable issue supporting the overall success of learners in higher education. Furthermore, building resilience in learners necessarily requires teachers’ efforts. Therefore, teacher support for autonomy is critical for augmenting appropriate outcomes, and it is deemed as a strong predictor of learners’ particular resources along with their motivational styles and educational achievement. As there is a dearth of studies that have considered teacher autonomy support and its noteworthy influence on learners’ resilience and engagement, the current review endeavors to concentrate on this motivational style in higher education. Successively, several implications are offered to illuminate the issue for teachers, students, teacher trainers, and educational administrators.

## Introduction

Resilience is a notion in the positive psychology literature that highlights institutions’ and people’s strengths and self-restraint to adapt to unexpected circumstances (Cooke et al., 2016). Likewise, it has been characterized as the capacity to accomplish constructive results regardless of openness to difficulty like trauma (Yoo et al., 2013). It is additionally characterized as the capacity of learners to successfully deal with academic decline, stress, and tension in the learning cycle (Sabouripour and Roslan, 2015). As stated by King (2004), the manifestation of connections that energize enthusiasm for proficient practice and self-comprehension could foster resilience, and also it can be influenced by both internal and external factors. The internal elements, such as self-confidence or good feelings are associated with what is perceived by the people, and their curiosity or grit in achievement (Zhou and Lam, 2019). In contrast to internal factors, external ones come from outside the person that can impact scholastic resilience, taken from family, qualified educators, peer relations, and the community or individual social climate (Everall et al., 2006). Resilience is similarly defined as an individual characteristic that is both intrapersonal and relational and arises dynamically from the transaction of various variables (Ungar, 2012; Turner et al., 2017). Similarly, it can be regarded as a context- and function-explicit notion which goes further than the preservation of equilibrium, being committed, and has agency (Gu, 2018).

Alongside resilience, learner engagement is one more developing field of interest inside the global education domain that is the main construct of positive psychology (Yu et al., 2019; Han and Wang, 2021; Wang et al., 2021). The relation between resilience and learner engagement is reported in some studies as students with low degrees of resilience also had low degrees of engagement (Pidgeon and Keye, 2014). One of the major problems for higher education in the 21st century is adequate learner engagement at the college level, as this idea identifies with quality assurance and improvement plans all over the world (Healey et al., 2014). Healey et al. (2014) maintained that learner engagement is an all-encompassing and multifaceted event that requires much more exploration to turn into a helpful policy for further developing learning in higher education. In the same vein, it has become essential to recognize the degree to which learners are involved and the successful instructive practices that strengthen engagement (Zepke, 2018; Kotera and Ting, 2019). Engagement, as aforementioned, is a dynamic and multi-layered attribute that can be influenced by different variables (Collins, 2014). Moreover, Guilloteaux (2016) grouped the impacting variables into phenomenological, individual-demographic, and informative variables.

People’s resilience and engagement depend on the individual, community-level, and organizational variables (Fernández-Martínez et al., 2017). One such relational variable that has been regarded to affect learners’ resilience significantly is social help. Some studies have demonstrated that social help might go about as a mediator between stress and resilience, while other studies demonstrated social help as relieving the adverse consequences of poor scholarly performance (Ozbay et al., 2007; London et al., 2011). A person with great social help can impact an individual’s ability to deal with upsetting experiences, adapt well to these experiences, and positively address these difficulties (Saam, 2010). Social help alludes to comfort, care, appreciation, or help accessible to a person from an individual/group (Sarafino and Smith, 2014) that can be arisen from different sources, like family, companions, and other people in their social surroundings (Permatasari et al., 2021). Concerning learners, they need a good amount of social help to strengthen their resilience when confronting pressing factors or stress (Ozbay et al., 2007).

The learner-educator frame explicitly calls attention to the fact that the connection between educators and learners plays the double role of developing or blocking learners’ student engagement and their learning motivation (Derakhshan et al., 2019; Pishghadam et al., 2021). Analysts have utilized an assortment of motivational principles to clarify the variables influencing student engagement, including the hypotheses of self-determination, self-guideline, objectives, as well as anticipated value (Fredricks et al., 2016). The Self-determination Theory (SDT) proposed that learners are all the more naturally motivated when educators support their fundamental mental requirements for autonomy, capability, and relatedness (Deci and Ryan, 2000). For elevating learners’ student engagement, a respectable educator-learner relationship is helpful in particular (Fredricks et al., 2016). In SDT, causal relations exist between mental requirements, motivation, engagement, and scholastic accomplishment (Reeve, 2012; Ryan and Deci, 2017).

What an individual does and says to determine, cultivate, and improve another person’s interior motivational assets is known as autonomy support (Reeve, 2009), which alludes to how much the social setting elevates practices started by and aligned with a person’s interests or wishes (Black and Deci, 2000). According to the SDT point of view, the volitional experience of motivation given to learners by an educator through class elements is known as teachers’ autonomy support (Deci and Ryan, 2000; Niemiec and Ryan, 2009). Teacher autonomy support is the provision of education through a relational nature of support (Assor et al., 2002; Reeve, 2016). Educators who use autonomy support as a relational educational style demonstrate less control and take better care of learners’ necessities, thereby increasing learners’ motivation and interest in the class (Chang et al., 2016; Pérez-González et al., 2019).

To examine how cultivating autonomy support permits the student to profit from various adaptive-related results, several studies have verified the utilization of SDT (Ryan and Deci, 2017). Based on their outcomes, individuals have an inborn inclination toward development. Moreover, they actively seek to deal with their current circumstance by making use of associations that permit them to incorporate new and positive encounters in elevating their sense of worth. Learners’ autonomy is increased when they are endorsed to regulate their conduct and when they believe that lessons are important to them (Wang and Eccles, 2016). Several examples of educator’s autonomy-supportive practices include paying attention to what learners need to say about the educational cycles, recognizing their viewpoint, empowering learners’ dynamic involvement, permitting learners to work in their specific manner, letting them control educational objects, conveying bases for learning capabilities, empowering independent work, and giving social awards to positive practices (Jang et al., 2010; Su and Reeve, 2011). Learners perceiving greater educator autonomy support report a more noteworthy feeling of competence, expanded self-guideline, commitment and scholastic motivations, and lower tension (Patall et al., 2010; Jang et al., 2012). Having additionally been discovered to upgrade learning-related results, like test performance for undergraduates, are autonomy-supportive class environments (Vansteenkiste et al., 2004). Through teacher autonomy support in their classroom, the learners’ basic psychosomatic needs for autonomy, competence, and relatedness are gratified, which consequently endorses their commitment in the classroom (Jang et al., 2016; Núñez and León, 2019). As stated by Reeve (2016), practically speaking, autonomy support includes a group of organized and mutually-strengthening educational practices, such as paying attention to learners’ point of view, vitalizing learners’ mental requirements during education, giving explanatory reasoning to educators’ demands, recognizing and tolerating learners’ demeanors of negative effect.

Learner academic achievement can be improved with autonomy-supportive teaching in higher education. An educational environment demands learners to be self-driven and self-determined which is more appropriate for students (Seli and Dembo, 2019). Learners who are encouraged to promote their autonomy are provided with choices, receive adequate support, and have the freedom to make their own learning decisions (Reeve and Jang, 2006). A bulk of research has proved the constructive effect of teacher autonomy-support on learners’ engagement (Chen et al., 2015; Hospel and Galand, 2016; Martin and Collie, 2019; Benlahcene et al., 2020; Li et al., 2020) and resilience (Reeve et al., 2020; Permatasari et al., 2021; Salazar-Ayala et al., 2021). Regardless of the abundance of research carried out on each construct so far on student engagement, resilience, and the function of teacher-autonomy support in higher education, one can notify plentiful studies each focusing on a specific concept. Nevertheless, to the best of the researcher’s knowledge, no studies to date have reviewed the aforementioned issue in learning and concurrently their association with each other has been not taken into consideration to date. In line with this aforementioned background, the present review sets about considering the issue.

## Literature Review

### Learner Engagement

Learner engagement refers to learners being enthusiastically engaged in the process of performing tasks (Lei et al., 2018). As stated by Chang et al. (2016), learners are interested in learning when they are participating in the educational system that is called engagement. Engaging learners on multiple levels, including intellectual, psychological, and emotional, is a multidimensional practice (Harbour et al., 2015; Datu, 2018; Xie and Derakhshan, 2021). The engagement has been explored from three different angles: educational, learner, and educator. From the educational context, engagement is defined as the amount of effort, care, resources, and skills that students apply to do tasks in classrooms and outside, and the methods and techniques educators use to encourage learners to engage with educational activities (Kuh, 2003). Behavioral engagement corresponds to the learners’ genuine disposition to participate in tasks (Fredricks et al., 2004; Bygate and Samuda, 2009). In recent studies, behavioral engagement has been viewed as learners’ participation during learning activities, their level of engagement, and how actively they are involved in educational processes (Hiver et al., 2021; Sang and Hiver, 2021).

Students’ psychological exertion and mental activity during the time spent on learning are known as cognitive engagement. Students are intellectually engaged when they display purposeful, specific, and maintained consideration to accomplish a given assignment or learning objectives (Reeve, 2012). Emotional engagement is viewed to have a significant effect on different elements of engagement on the grounds that the abstract mentalities or discernments students convey in a class or through related assignments are basic to different elements of engagement (Henry and Thorsen, 2020). Thus, affective (emotional) engagement alludes to students’ perspectives toward the learning settings, the individuals in that unique setting, the assignments, and their cooperation in education (Skinner et al., 2009; Reeve, 2012). As asserted by Philp and Duchesne (2008), social engagement holds a focal spot in language learning; indeed, the social part of engagement is characterized by considering the societal types of tasks and contributions that are perceptible in networks of learning, including association with questioners as well as the nature of such social connections (Linnenbrink-Garcia et al., 2011; Mercer, 2019).

### Self-Determination Theory

An organismic-argumentative point of view that characterizes individuals as proactive beings who are inherently prompted to develop, work, and change inside their social environments is known as SDT (Deci and Ryan, 2000). SDT states that people have three major requests, namely, the necessity for autonomy, the requirement for capability, and the requirement for belongingness. How educators meet learners’ essential requirements will impact the latter’s prosperity, motivation, engagement, and accomplishment (Ryan and Deci, 2017; Núñez and León, 2019). SDT proposes that, aside from physical requirements like food and shelter, essential mental requirements of autonomy, relatedness, and capability are key assets on which an individual is reliant for flourishing (Ryan and Deci, 2020). As stated by Deci and Moller (2005), the requirement of competence is characterized in SDT as the sense of value, capacity, and accomplishment in one’s communications inside a societal climate. Feelings of capability are experienced in settings that furnish people with promising circumstances and assets to communicate, enhance, and ace their abilities (Ryan and Moller, 2016). As declared by Deci and Ryan (2000), the feeling of connectedness or belongingness to an individual or a specific group is known as the requirement of relatedness (Deci and Ryan, 2000) that tends to be satisfied only when relationships are autonomous and genuine to oneself and others (Ryan and Deci, 2017). “Autonomy” is characterized as self-administering or alluded to as self-guideline, which is the method of self-directing one’s practices and activities (Ryan and Deci, 2020). Because of its vital function in the fulfillment of different necessities, the requirement of autonomy has attained significant consideration. Moreover, it is portrayed by SDT researchers as a sense of preference, whereby one’s activities are coordinated by oneself or self-supported as opposed to being externally controlled (Ryan and Deci, 2017).

### Autonomy Support

Autonomy is attained from experiences and practices that are regarded as self-controlled, self-embraced, and are lined up with people’s actual qualities and interests (Ryan and Deci, 2017). Practices and mentalities that are experienced through relational association can therefore be autonomy-supportive when they are regarded as advancing self-controlled decisions and motivations (Reeve, 2009; Ryan and Deci, 2017). In young learners, autonomy support has been positively connected with self-controlled learning, profound data processing, persistence in defining and meeting objectives, higher scholastic performance, and well-being, and less tension in students (Kins et al., 2009; Kunst et al., 2019). As declared by Niemiec and Ryan (2009), inside the domain of academia, autonomy support elevates intrinsic motivation which, thus, improves learners’ learning, change, and performance in scholastic assignments (Niemiec and Ryan, 2009). Accordingly, autonomy support is a critical factor in the internalization and the quest for instructive objectives and scholastic performance. Three primary kinds of autonomy support have been proposed by Stefanou et al. (2004), namely, intellectual, procedural, and institutional. Intellectual autonomy support includes strategies that empower learners to have an independent mind, explore thoughts, and become independent students. Enhancing learners’ ownership of form and presentation is procedural autonomy support. Institutional autonomy support empowers learners’ ownership of the educational climate. Educators might utilize at least one type of these techniques, but a few researchers have proposed that, due to its function of cultivating learners’ mental engagement and profound-level processing, intellectual autonomy support might be the most advantageous (Assor et al., 2002; Stefanou et al., 2004; Wang and Guan, 2020).

### Resilience

Even though there is no concurred meaning, resilience is usually viewed as a paradigm, where internal assets and practices are encompassed to adapt to troubles and difficulties, thereby prompting a reinforced character and mental coping mechanism (Grant and Kinman, 2014). By reshaping one’s viewpoints, resilience makes one pay attention to strengths and chances, as opposed to shortcomings and weaknesses (Russ et al., 2009; Harrison, 2013). Resilience can likewise be seen as a trait evolving from a resilient structure and contains three sets of associating constructs, in particular, internal assets, external help systems, and learned methods (Kostoulas and Lämmerer, 2018). Resilience, in the teaching profession, is an imperative key to comprehend both educating and learning procedures (Hui and Abdullah, 2020; Xue, 2021) and takes place when people link their assets with context-oriented ones and utilize successful techniques to overcome hardships and maintain their well-being (Greenier et al., 2021). In the territory of teaching and learning a language, resilience is a new issue that has been well-defined as a stress-managing aptitude (Connor and Davidson, 2003). In general, resilience is regarded as a persons’ ability to rebound back from hardships and adjust to their setting as Martin and Marsh (2006) referred to resilience as a learner’s capability to effectively cope with obstructions, contests, difficulty, and burden in the theoretical situation.

## Implications and Future Directions

In light of this review, evidence is proved for the significance of autonomy support and its academic benefits in higher education. An important implication for teachers is that they should endeavor to represent autonomy-supportive instruction that is useful for learners in higher education. When educators establish a powerful class climate and allow learners to work following their arising interest and coordinated value, they can assist their learners with satisfying their requirement of autonomy, thereby developing self-determined activities. Thus, in an autonomy-supportive class climate, learners have greater interest, more noteworthy energy, better relatedness, and less pressure. Furthermore, teacher autonomy support can be deemed as a learner-centered approach that arranges for the required support for teachers to increase their motivating styles. It is likely that during autonomy support, when the students are provided with clues and feedback, and they are praised by the teacher, they tried more to comprehend their task better and learn the lesson.

When the students are taught by autonomy-supportive teachers, positive academic outcomes are achieved including higher resourcefulness, more satisfaction, and determination, positive reactions, and enthusiasm that all necessary for their engagement in the process of learning and enhances their resilience because when teachers detect learners’ desires, inclinations, and interests, bring them about by boosting and cultivating satisfactory classroom settings (Reeve and Jang, 2006; Hang et al., 2017). Educators are recommended to carry out autonomy support TAS by listening cautiously to learners and recognizing their point of view, giving them chances for dynamic participation, permitting them to work in their favored way, permitting learners to control educational objects, conveying a reasoning learning, providing encouragement, and praising as a reward (Kaur et al., 2014). Through autonomy support, the teacher can increase autonomy opportunities, which can be considered as an operative way of decreasing apprehension and depression in learners (Yu et al., 2016).

In particular, teacher autonomy support essentially elevates the requirement of autonomy, capability, and relatedness, accordingly making teenagers more engaged in and connected with their academic day-by-day activities. Thus, this upgraded academic engagement mitigates or counterbalances adolescent stress and sadness. Academic engagement is enhanced by fundamental mental necessities satisfaction, which leads to a decreased probability of stress and sadness. As a kind of autonomy-supportive practice to determine learners’ mental necessities and incorporate them into the day’s lesson, the educator can ask learners what they need. Another kind of autonomy-supportive practice is educators giving learners time to solve an issue in their specific way since the educator permits learners’ interests and inclinations to direct their class activity. The educator who upholds autonomy is not only increasing the number of autonomous learners interested in the class but is also contributing to their mental and add social well-being, enhancing and expanding an adaptive and resilient practice despite age-related afflictions and others brought about by outer components (Salazar-Ayala et al., 2021). Using autonomy support as a relational educational style, educators exhibit less control and take care of learners’ requirements, which can enhance learners’ motivation and interest in their classes that prompt their engagement (Núñez and León, 2019).

The outcomes of the research could be useful for instructive psychologists, counselors, educators, instructive researchers, and educational program designers to put together a few projects to upgrade the adapting and resilience level of learners, which directly affects their presentation and educational level. Generally, fostering resilience through autonomy support has significant implications in psychology in five principal roles, namely, evaluation, mediation, discussion, research, and preparation. Moreover, through this review, social and psychological researchers and learners may be likewise provided with certain attributes of character and practices among people who should be searched further.

Great degrees of social support are connected with both high resilience and more prominent psychological well-being (Bovier et al., 2004). Indeed, the execution of casual perceived social support intercessions inside the instructive climate might be altogether useful in developing resilience, enhancing the psychological health of learners, and possibly elevating learners’ retention. Social support has positive impacts on students’ engagement and resilience, demonstrating that the education staff ought to urge their educators to partake in society or institutional exercises or even practice their plans through remuneration frameworks to improve educators’ contribution motivation and reinforce friendly bonds and supports by setting up rich and diverse companion-level social communities to offer adequate help when needed.

In addition, educational faculty could hold courses and workshops covering the hypothetical and pragmatic parts of emotional and educational support for educators to improve their capacity to offer support. Subsequently, students’ engagement and resilience can be elevated by building up others’ capability to give needed help and support in various circumstances. To assist educators with understanding the advantages of autonomy support and implement it in their training, educator trainers should attempt to design projects. Completely developed and versatile autonomy-support training plans should be made promptly available to schools and educators. Besides, administrators and managers can create a more extensive context of a strategy that leads to educators feeling reinforced in their requirements of autonomy, capability, and relatedness; therefore, they are rendered to support the requirements of their learners. Further qualitative studies must be led for surveying the collaboration of various variables among learners to reach a more profound comprehension of the notion of resilience. Thus, in future investigations, it is recommended that analysts utilize longitudinal studies to uncover cause-and-effect relationships among factors. One more significant outcome of this contribution is the significance of autonomy support provided by educators in the social climate in which learners are involved. Expansion of the current research would be to explore the relationship of perceived autonomy support from educators with different indicators for college learners’ scholarly change, like perspectives and observed control (Hagger and Chatzisarantis, 2016; Respondek et al., 2017).

## Author Contributions

The author confirms being the sole contributor of this work and has approved it for publication.

## Conflict of Interest

The author declares that the research was conducted in the absence of any commercial or financial relationships that could be construed as a potential conflict of interest.

## Publisher’s Note

All claims expressed in this article are solely those of the authors and do not necessarily represent those of their affiliated organizations, or those of the publisher, the editors and the reviewers. Any product that may be evaluated in this article, or claim that may be made by its manufacturer, is not guaranteed or endorsed by the publisher.
